# Retrospective analysis of factors that affect the success of single-dose methotrexate treatment in ectopic pregnancy

**DOI:** 10.4274/tjod.10576

**Published:** 2015-12-15

**Authors:** Altan Var, Ramazan Özyurt, Bulat Aytek Şık, Serkan Kumbasar, Erman Sever, Mustafa Deveci, Özgür Çöt, Süleyman Salman, Yılmaz Güzel

**Affiliations:** 1 İstanbul Research and Education Hospital, Clinic of Obstetrics and Gynecology, İstanbul, Turkey; 2 İstanbul Aydın University Faculty of Medicine, Department of Obstetrics and Gynecology, İstanbul, Turkey; 3 Sakarya University Faculty of Medicine Research and Education Hospital, Department of Obstetrics and Gynecology, Sakarya, Turkey; 4 Gaziosmanpaşa Taksim Research and Education Hospital, Clinic of Obstetrics and Gynecology, İstanbul,Turkey

**Keywords:** Ectopic pregnancy, Methotrexate, β-hCG

## Abstract

**Objective:**

Detection of factors that affect the success of single-dose methotrexate treatment in ectopic pregnancy.

**Materials and Methods:**

We investigated 99 patients who had been treated with single-dose methotrexate for ectopic pregnancy in our clinic between January 2009 and June 2014. Demographic, clinical, and laboratory results of possible factors that affect treatment success were retrospectively analyzed. Successfully and unsuccessfully treated patients were compared based on their pre-treatment results.

**Results:**

The success rate of single-dose methotrexate treatment was found to be 70.7%. No significant difference was found between succesfully and unsuccessfully treated patients before treatment in terms of factors such as gestational weeks, mass size, presence of yolk sac, and presence of free fluid (p=0.224, p=0.201, p=0.200, p=0.200). Serum β-hCG values in patients whose treatment was unsuccessful was found to be higher compared with the successfully treated group (mean β-hCG value of unsuccessful group: 4412±3501 mIU/mL; mean β-hCG value of successful group: 1079±942 mIU/mL; p<0.001).

**Conclusion:**

Single-dose methotrexate treatment is an effective and reliable method in the treatment of ectopic pregnancy. Elevation of serum β-hCG value stands as the main prognostic factor that affects the success of single-dose methotrexate treatment.

## INTRODUCTION

The incidence of ectopic pregnancy, which is defined as the implantation of fertilized ovum out of endometrial cavity, varies between 1-16/1.000 among all pregnancies^(1)^. Tubal surgery, infection or congenital tubal damage, pelvic inflammatory disease (PID), presence of intrauterine device (IUD), and previous ectopic pregnancies are the most significant risk factors^(2)^. The chances of early diagnosis have increased and mortality associated with ectopic pregnancy have decreased from 19.6/10.000 to 3.4/10.000 because transvaginal ultrasonography and sensitive laboratory β-hCG measurements have become common place^(3)^. Along with the traditional surgical treatment for ectopic pregnancy, today there are several medical and expectant treatment options. Methotrexate (MTX) is used as single dose in eligible patients, repeat doses are performed when necessary^(4)^. In patients with ectopic pregnancy that have not ruptured, this treatment is preferred due to its high success and low complication rates, fewer adverse effects, and less loss of labor^(5)^.

The aim of the study was to investigate prognostic factors that affect the success of single-dose MTX treatment in patients who were diagnosed as having an ectopic pregnancy and underwent medical treatment with single-dose MTX within a ten-year period in our clinic.

## MATERIALS AND METHODS

A total of 182 pateints who were diagnosed as having an ectopic pregnancy and were treated medically or surgically between January 2010 and June 2014 in İstanbul Research and Training Hospital Maternity Clinic were retrocpectively evaluated. Criteria for ectopic pregnancy diagnosis were determined as serum β-hCG above 1.500 IU/mL, absence of intrauterine pregnancy sac in transvaginal ultrasonography, detection of an abnormal increase in β-hCG follow-ups (increase less than 60% in values checked at 48 hours) or endometrial curettage pathology. The patients’ files and operation reports were examined and the following characteristics were recorded, age, number of pregnancies, delivery, abortion and curettage, contraception methods, previous ectopic pregnancies, tubal sterilization history, serum β-hCG value at admission, ultrasonographic findings, and treatment types. Seventy patients who underwent emergency or elective surgical procedures as the starting treatment and 13 patients who underwent tubal abortion or spontaneous resolution through the waiting approach were excluded from the study. Ninety-nine patients who were planned to have single-dose MTX treatment, who were hemodynamically stable, and who did not present with fetal cardiac activity and rupture findings in transvaginal ultrasonography were included. MTX treatment was used in the absence of MTX allergy, and liver, lung, kidney, and hemotologic diseases. To detect this, blood groups, complete blood counts, liver function tests, blood urea nitrogen, and creatinine levels were measured. Patients with a histroy of lung disease were evaluated with lung graphy because of the risk of intersistitial pneumonitis associated with MTX. Informed consent was taken from the patients for MTX treatment. MTX was given to eligible patients as a single 50 mg/m^2^ intramuscular dose. Rh immunoglobulin was given to the patients who had Rh incompatibility. The day of MTX administration was accepted as the first day. Serum β-hCG values were recorded on the first day. β-hCG values were repeated on the 4^th^ and 7^th^ days. All patients’ β-hCG values were assessed weekly for the presence of a decrease more than 15% between β-hCG values between the 4^th^ and 7^th^ days; a decrease in β-hCG value below 25 IU/mL was evaluated as successful treatment. Patients whose β-hCG values decreased less than 15% between the 4^th^ and 7^th^ days were evaluated as not successful after single-dose MTX treatment. These patients received a second dose of MTX. Development of hemodynamic stability or preference for surgery for any reasons were considered as treatment failure. Statistical analyses of the study was performed using the Statistical Package for Social Sciences software (SPSS) version 18.0. Chi-square and Fisher’s exact test were used for the comparison of variables. P<0.05 was considered as statistically significant.

For the evaluation of independent associations of each predicting factor (presence or absence of yolk sac, free peritoneal fluid, and pseudogestational) with following response of patients to MTX, multiple logistic regressions were used. Differences were considered statistically significant when the p value was<0.05.

## RESULTS

A total of 182 pateints who were diagnosed as having an ectopic pregnancy and treated between January 2004 and June 2014 were subjected to evaluation. Ninety-nine patients who met the study criteria and enrolled in the study had been given single-dose MTX as the starting treatment.

Although single-dose MTX treatment was successful in 70 patients (70.7%), it was unsuccessful in 29 (29.2%) patients. The demographic, clinical, and laboratory findings of the patients whose treatment with single-dose MTX was successful and unsuccessful are shown in Tables 1 and 2. There were no significant differences between groups in terms of age, parity, abortus, gestational weeks at admission, ectopic pregnancy mass size, presence of pseudogestational sac or yolk sac, and presence of free fluid.

Although treatment was successful in 15 (21.4%) of 19 patients who were given single-dose MTX and had a yolk sac at transvaginal ultrasonography, 4 patients (13.7%) did not respond to treatment (p=0.210). Similarly, treatment was susccessful in 22 of 30 patients who presented with a pseudogestational sac; this number constituted 31.4% of all patients whose MTX treatment was successful (Table 3).

**Figure 1:**
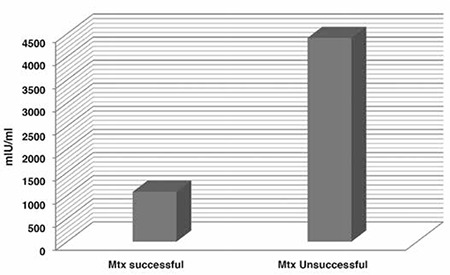
Mean β-hCG values in the groups that underwent successful and unsuccessful single-dose methotrexate treatment (mIU/ml)

The mean β-hCG value of the successful MTX group was 1079±942 mIU/mL and the median β-hCG value was 810 mIU/mL (range, 131-3730 mIU/mL); the mean β-hCG value in the unsuccessful MTX group was 4412±3501 mIU/mL and the median β-hCG value was 3694 mIU/mL (range, 630-9600 mIU/mL). Based on these data, serum β-hCG values in the unsuccessful MTX group was found higher than in the successful MTX group (Figure 1).

The sensitivity and specificity of these variables as well as the optimal cut-off points were calculated using an receiver operating characteristic curve. For baseline hCG levels, the optimal cut-off point was 2.483, the sensitivity was 80% and specificity was 70%. Using these cut-off values, the odds ratios of these variables were then calculated. For a baseline hCG level 2.483 mIU/mL, an OR of 0.08 and a p value<0.01 was found (95% confidence interval (95% CI) 0.02-0.32), demonstrating a probability of 92% of achieving therapeutic success with MTX. Regression analysis revealed the pretreatment serum chorionic gonadotropin concentration to be the only factor that contributed to the success rate. The presence or absence of yolk sac, free peritoneal fluid, and pseudogestational sac were not found to be statistically significant predictors of success in our review (Table 4).

## DISCUSSION

Today, surgical, medical, and conservative approaches are performed in the treatment of ectopic pregnancy, which is one of the most common causes of first trimester maternal morbidity^(6,7)^. Development of additional diagnostic methods used for early diagnosis of ectopic pregnancy have led to a more common use of conservative surgical treatment. The goal in the treatment of ectopic pregnancy has now been directed to preservation of fertility rather than saving lives. The more common use of MTX for early diagnosis and treatment of ectopic pregnancy has decreased the incidence of surgical treatment^(8)^.Stowall and Ling^(4)^ studied the efficiency of a systemic use of single-dose MTX in 1993. All patients were diagnosed using a non-laparoscopic algorithm such as serial β-hCG titers, serum progesterone levels, transvaginal ultrasonography, and dilatation and curettage. One hundred twenty patients who had haemodynamically stable and nonruptured ectopic pregnancy not more than 3.5 cm were given 50 mg/m^2^ single-dose intramuscular MTX. Complete resolution was provided in 113 patients (94.2%) within a mean of 35.5 days. Four (3.3%) patients who were successfully treated required a second MTX dose on the 7^th^ day. Seven (5.8%) patients required a surgical approach, two of whom presented with cardiac activity^(7)^. Stovall et al.^(9)^ published the outcomes of single-dose MTX treatment in 100 patients in 1991. Fifty of 100 patients were diagnosed with laparoscopy, the other 50 were diagnosed with a non-laparoscopic algorithm. Complete resolution was provided in 96 patients within 14-92 days. Laparotomy was required in 4 patients due to tubal rupture; rupture occurred in one case at a late stage on the 23rd day of MTX treatment. Fetal cardiac activity was observed in 5 patients and treatment was successful in all these patients^(9)^. Success rates of single-dose MTX use, which is an effective and reliable treatment option in ectopic pregnancy, varies between 64% and 94.2%^(10)^. In our study, the success rate was found to be 70.7%, similar to other studies in the literature. Nineteen out of 29 (29.2%) patients whose treatment was unsuccessful were given a second dose of MTX, and 10 patients underwent surgical treatment. Eight patients underwent unilateral salpingectomy and unilateral salpinoopherectomy was performed in 2 patients who required emergency laparotomy because of tubal rupture.

In the study by Alkış and Mungan.^(11)^ success was provided in 81 (82.7%) of 98 patients with single-dose MTX; it was unsuccessful in 17 patients, all of whom underwent surgery. The patients’ mean initial β-hCG values were found to be 1592±2613 mIU/mL. In the study by Adalı et al.^(12)^ single-dose MTX treatment was successful in 14 (77.7%) of 18 patients. Initial β-hCG levels were detected as 2615±2064 mIU/mL. Surgery was performed in 4 (22.3%) patients whose MTX treatment was unsuccessful. In our study, initial serum β-hCG levels were found as 2037±2070 mIU/mL. The mean β-hCG level in the successful group was 1079±942 mIU/mL; the mean β-hCG value in the unsuccessful group was 4412±3501 mIU/mL. According to this, serum β-hCG values of the unsuccessful group were higher compared with the successful group (p<0.05).

Lipscomb et al.^(13)^ published a review about successful treatment of ectopic pregnancy with single-dose MTX in 1999. In this review, logistic regression analyses revealed that serum β-hCG levels before treatment was the only factor that affected treatment failure. It was indicated that size and volume of the mass, presence of hematoma, size of hematoma if present, and presence of free fluid in the pelvis were not significant risk factors for treatment failure. In our study, it was detected that among the parameters of age (p=0.300), gravida (p=0.210), gestational weeks at diagnosis (p=0.224) and mass size before treatment (p=0.201), only serum β-hCG values before treatment were detected to be a factor that affected treatment success (OR: 0.08; p<0.001).

Pre-treatment β-hCG values were used as the determinant of systemic single-dose MTX treatment in most of the published studies. According to Lipscomb et al.^(14)^ serum β-hCG values were the most significant factor in the success of single-dose MTX treatment.

Reports regarding prognostic factors of successful responses to single-dose MTX treatment in ectopic pregnancies are limited. Although high levels of serum β-hCG were detected as the only effective prognostic factor in many studies, as in our study,^(9,15)^ Kimiaei et al.^(16)^ found that ectopic focus size was also important, in addition to initial β-hCG values in determining the efficiency of single-dose MTX treatment. Moreover, it was stated in some studies that the initial and level of decrease in β-hCG values on the 4^th^ day following MTX were the most significant indicators in predicting success in addition to the increase in the initial β-hCG value^(17)^. In the study by Mungan et al.^(18)^ it was shown that settlement of ectopic pregnancy on tuba uterina was also important in determining the success of single-dose MTX treatment in ectopic pregnancy. Single-dose MTX treatment was successful in 73 (74.4%) of 98 patients; however, no dramatic difference was observed in the success rates when patients were grouped based on periampullary and periisthmic settlement. Although success was provided in 77 (91.6%) of 84 patients with periampullay settlement, treatment was found to be successful in 4 (28.5%) of ectopic pregnancies with periisthmic settlement.

In a 2005 study by Bixby et al.^(19)^ it was shown that presence of yolk sac in ectopic gestational sac or presence of fetal heart beat negatively affected the success of treatment, besides high β-hCG. In a study that evaluated 62 patients, single-dose MTX treatment was ended with failure in 1 patient (1.5%) who presented with a fetal heart beat, and 15 patients (24%) who had a yolk sac (p<0.05). In the same study, it was shown that presence of pseudogestational sac (p=0.200) and presence of free fluid in the pelvis (p=0.200) did not affect the success of the treatment. It was also detected in our study that presence of pseudogestational sac (p=0.352), presence of free fluid in the pelvis (p=0.200), and the presence of yolk sac (p=0.200) had not affect on treatment success.

In conclusion, ectopic pregnancy, which has an increasing incidence today, is one of the most significant causes of maternal mortality and morbidity^(20)^. Single-dose MTX treatment is an effective treatment in the initial treatment of ectopic pregnancy. Success rates vary between 64% and 94.2% in the literature^(21)^. In the present study, we showed that the increase in initial serum β-hCG values was the most significant factor to affect the success of single-dose MTX treatment in ectopic pregnancy. We found that factors such as ectopic pregnancy focus size, presence of yolk sac, presence of free fluid, age of the mother, and gestational weeks had not affect on treatment success. It is possible to detect prognostic factors in MTX treatment and to increase success rates of treatment in the future by investigating these and similar prognostic factors in many more studies.

## Figures and Tables

**Table 1:**
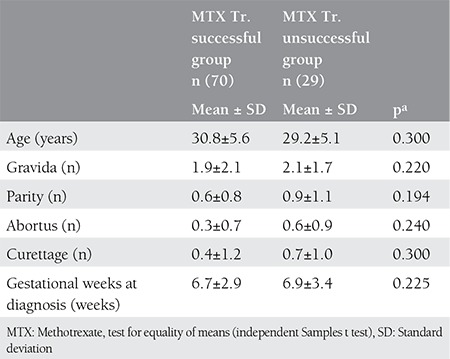
Demographic and clinical characteristics of the patients groups who succeded and did not succeed with single-dose methotrexate treatment

**Table 2:**
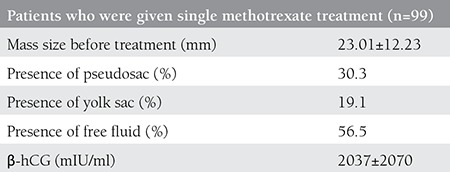
Transvaginal ultrasonography and laboratory results of patients who were given single-dose methotrexate treatment

**Table 3:**
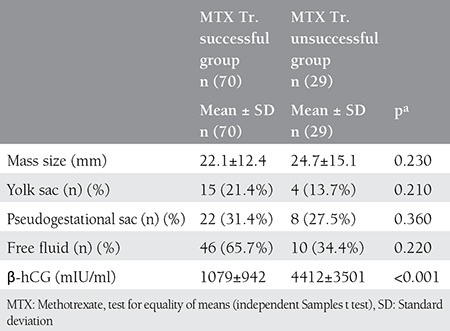
Ultrasonographic and laboratory results of patient groups whose single dose methotrexate treatment was successful and unsuccessful

**Table 4:**
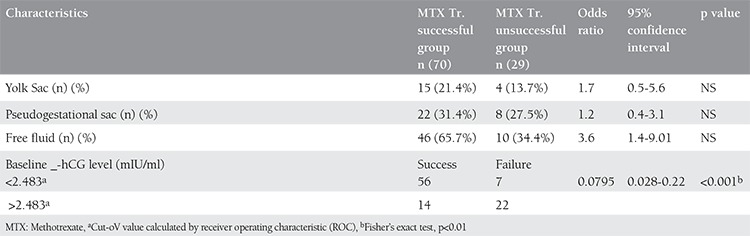
Logistic regression for the comparison of groups according to success or failure of therapy

## References

[1] Berek Jonathan S (2007). Berek & Novak’s Gynecology, Lippincott Williams & Wilkins 1. Publications. 14th Edition.

[2] no author (2007). Keith Edmonds Dewhurst’s. Textbook of Obstetrics & Gynaecology, Blackwell Publishing.

[3] Coste J, Job-Spira N, Aublet-Cuvelier B, Germain E, Glowaczower E, Fernandez H, et al (1994). Incidence of ectopic pregnancy. First results of a population-based register in France. Hum Reprod.

[4] Stovall TG, Ling FW (1993). Single-dose methotrexate; an expanded clinical trial. Am J Obstet Gynecol..

[5] Alper AGO, Büyükbayrak EE, Bayramoğlu MB, Karşıdağ AYK, Kars B, Pirimoğlu ZM, et al (2010). Ektopik gebelikte tedavi yaklaşımları: Tersiyer bir merkezin 4 yıllık retrospektif analizi. Turkiye Klinikleri J Gynecol Obst.

[6] Muresan D, Stamatian F, Ona D, Cruciat G, Caracostea G, Rotar I, et al (2008). Current trends in the treatment of ectopic pregnancy. Chirurgia (Bucur).

[7] Mol F, Mol BW, Ankum WM, Hajenius PJ (2008). Current evidence on surgery, systemic methotrexate and expectant management in the treatment of tubal ectopic pregnancy: A systematic review and meta-analysis. Hum Reprod Update.

[8] Murray H, Baakdah H, Bardell T, Tulandi T (2005). Diagnosis and treatment of ectopic pregnancy. CMAJ.

[9] Stovall TG, Ling FW, Gray LA (1991). Single-dose methotrexate for treatment of ectopic pregnancy. Obstet Gynecol.

[10] Loi K, Wang J J, Siow A (2009). Methotrexate treatment for ectopic pregnancy at the KK Women’s and Children’s Hospital, Singapore. Singapore Med J.

[11] Alkış İ, Mungan T (2009). Dış gebelikte 50 mm/m2 IM tek doz methotrexat tedavisi. J Turk Soc Obstet Gynecol.

[12] Adalı E, Kurdoğlu M, Kolusarı A, Yıldızhan R, Çim N, Şahin HG (2010). Kliniğimizdeki ektopik gebelik olgularının beş yıllık analizi. J Turk Soc Obstet Gynecol.

[13] Lipscomb GH, McCord ML, Stovall TG, Huff G, Portera SG, Ling FW (1999). Predictors of success of methotrexate treatment in women with tubal ectopic pregnancies. N Engl J Med.

[14] Limbscomb GH, Stovall TG (2000). Non surgical treatment of ectopic pregnancy. New Engl J Med.

[15] Yıldırım G, Güngördük K, Aktaş FN, Ülker V, Sudolmuş S, Tekirdağ Aİ (2007). Ektopik gebelik tedavisinde tek doz metotreksat: 85 olgunun değerlendirilmesi. J Turk Soc Obstet Gynecol.

[16] Kimiaei P, Khani Z, Marefian A, Ghavamabadi MG, Salimnejad M (2013). The importance of gestational sac size of ectopic pregnancy in response to single-dose methotrexate. ISRN Obstet Gynecol.

[17] Stika CS, Anderson L, Frederiksen MC (1996). Single-dose methotrexate fort he treatment of ectopic pregnancy: Northwestern Memorial Hospital three-year experience. Am J Obstet Gynecol.

[18] Mungan T, Erdemoğlu E, Guney M (2007). The effect of the ultrasonographically determined tubal implantation site in ectopic pregnancy on the success of methotrexate treatment and subsequent reproductive outcome. J Turk Ger Gynecol Assoc.

[19] Bixby S, Tello R, Kuligowska E (2005). Presence of a yolk sac on transvaginal sonography is the most reliable predictor of single-dose methotrexate treatment failure in ectopic pregnancy. J Ultrasound.

[20] Berg CJ, Chang J, Calloghan WM, Whitehead SJ (2003). Pregnancy-related mortality in the United States, 1991-1997. Obstet Gynecol.

[21] Fernandez H, Lelaidier C, Thouvenez V, Frydman R (1991). The use of a pretherapeutic, predictive score to determine inclusion criteria for the non-surgical treatment of ectopic pregnancy. Hum Reprod.

